# Motor Evoked Potentials in Hereditary Spastic Paraplegia—A Systematic Review

**DOI:** 10.3389/fneur.2019.00967

**Published:** 2019-09-18

**Authors:** Sue-Faye Siow, Ruaridh Cameron Smail, Karl Ng, Kishore R. Kumar, Carolyn M. Sue

**Affiliations:** ^1^Department of Neurogenetics, Kolling Institute, Royal North Shore Hospital, St Leonards, NSW, Australia; ^2^Department of Neurology and Neurophysiology, Royal North Shore Hospital, St Leonards, NSW, Australia; ^3^Northern Clinical School, Kolling Institute, Royal North Shore Hospital, St Leonards, NSW, Australia; ^4^Sydney Medical School, University of Sydney, Sydney, NSW, Australia; ^5^Department of Neurology, Concord Hospital, Sydney, NSW, Australia; ^6^Kinghorn Centre for Clinical Genomics, Garvan Institute of Medical Research, Darlinghurst, NSW, Australia; ^7^Molecular Medicine Laboratory, Concord Hospital, Sydney, NSW, Australia

**Keywords:** hereditary spastic paraplegia, motor evoked potentials, systematic review, biomarker, clinical trials

## Abstract

**Background:** Hereditary Spastic Paraplegia (HSP) is a slowly progressive neurodegenerative disorder with no disease modifying treatment. Potential therapeutic approaches are emerging and large-scale clinical drug trials for patients with HSP are imminent. A sensitive biomarker to measure the drug efficacy in these trials is required. Motor evoked potentials (MEPs) are a potential biomarker for HSP as they assess the central motor pathways and can be standardized with set protocols and guidelines.

**Objectives:** We performed a systematic review to investigate the utility of MEPs as a diagnostic and disease severity biomarker for HSP.

**Search Methods:** Systematic searches of PubMed, Embase, Medline, and Scopus were performed.

**Selection Criteria:** Studies reporting on central motor conduction time measured with MEPs in adult and pediatric patients with HSP were included. We excluded studies in non-HSP patient cohorts, not in English, not original research, and unpublished journal articles.

**Data Collection and analysis:** Search results were de-duplicated and screened according to the inclusion and exclusion criteria. The included papers were reviewed independently by two reviewers and data was collected on patient cohorts, test methods, results, and study quality. Results were analyzed using descriptive methods.

**Results:** Of the 882 search results, 32 studies were included in the review. The most common finding was absent or prolonged lower limb (LL) central motor conduction time (CMCT) in patients with HSP (78% of patients studied). Quality assessment revealed variability in study methodology and reporting of results. Variations included patient cohorts of various genotypes as well as variations in equipment and techniques used. Aside from CMCT, none of the MEP parameter measures correlated with disease severity and many did not show significant difference between HSP patients and controls.

**Conclusion:** Systematic review of MEP studies in HSP patient cohorts demonstrated mixed findings. Lower limb CMCT was the most promising parameter in terms of differentiating HSP patients from controls, with one study demonstrating a weak correlation with clinical disease severity. It is possible that the lack of consistency in study methodologies and small patient cohorts have contributed to the variable findings. A longitudinal study of MEPs in a large cohort of HSP patients with the same genotype will help clarify the utility of MEPs as a biomarker for disease severity and use in clinical trials.

## Introduction

Hereditary spastic paraplegia (HSP) encompasses a group of neurodegenerative conditions that result in lower limb spasticity and weakness. Despite causing significant disability, there is no available cure for this progressive condition (1). The underlying pathophysiology of HSP remains poorly understood and it is likely that this varies according to genotype (2). There are >64 HSP associated genes and 13 HSP associated loci identified to date and this number will continue to grow with the advent of next generation sequencing (3, 4).

Recent research using stem cell models to study HSP disease pathogenesis, including SPG4 (HSP-*SPAST*), SPG3A (HSP-*atlastin-1*), and SPG11 (HSP-*spatacsin*) (5–7), have revealed several potential treatment options targeting the underlying disease mechanisms (8). There is a paucity of clinical trials for HSP drug treatment options, the most recent trials targeting SPG5 (HSP-*CYP7B1*) showed improvement of biological markers but no discernible clinical improvement (1, 9, 10).

Biomarkers of disease severity and progression are vital components of establishing a clinical trial for therapeutic agents in HSP. Although several different biomarkers have been used in the small number of clinical trials published to date, a standardized biomarker for use across all clinical trials has not been defined (9–11). Promising biomarkers for disease severity in HSP include the Spastic Paraplegia Rating Scale (SPRS) (10), gait analysis (12), motor evoked potentials (MEPs) (13), diffusion tensor imaging (DTI) (14), and genotype-specific biochemical markers, such as 27-hydroxycholesterol in SPG5 (9, 10). Ideally, a disease biomarker for HSP should be easily accessible, able to be standardized across different institutions, affordable and minimally invasive. Challenges to developing a standardized biomarker include heterogeneity of HSP genotypes, broad spectrum of associated features and the typically slow progression of HSP.

Motor evoked potentials (MEPs) elicited through transcranial magnetic or electric stimulation have been proposed as a biomarker for disease severity in HSP (13). Prolongation of the central motor conduction time (CMCT) is used as a marker for upper motor neuron abnormalities causing slowing of conduction (15). There are clear guidelines for measurement of MEPs allowing standardization across multiple centers, a useful feature for a disease biomarker (16). In order to evaluate the utility of MEPs as a biomarker for HSP, we performed a systematic review on the measurement of MEPs in HSP patient cohorts. Our aim was to evaluate CMCT as a diagnostic tool and as a biomarker for disease severity in HSP.

## Methods

We performed a systematic search of PubMed, Embase, Medline and Scopus. The full search terms are included in Appendix 1. In short, the terms used were “hereditary spastic paraplegia” AND [“motor evoked potentials” OR “transcranial magnetic stimulation” OR “central motor conduction time”].

The search results were exported to EndNote and de-duplicated. The first screen was of the titles and abstracts according to the inclusion and exclusion criteria. The second screen was of the full article. The included studies were then reviewed independently by two reviewers.

Inclusion criteria were full text articles only, English articles, humans only.

Exclusion criteria were conditions that were not hereditary spastic paraplegia, articles that did not measure central motor conduction time using transcranial stimulation, not original research.

Data were collected on patient demographics, neurophysiological techniques, and study results using a pre-set form that populated a database (Appendix 2). Assessment of methodological quality was performed with the NIH Study Quality Assessment Tool (17), scored out of 12 points for case control studies and 9 points for case series; as well as a 24 point checklist for assessing methodological quality of transcranial magnetic stimulation studies (18) (Appendix 3). Any discrepancies between the reviewers were resolved with discussion and if required, a third reviewer was involved.

Data was analyzed with descriptive methods and presented in tables and graphs. Meta-analysis could not be performed due to the differing methodologies and heterogeneity of the results reported by the studies.

## Results

### Search Results and Screening Process (PRISMA Diagram)

**Figure 1:**
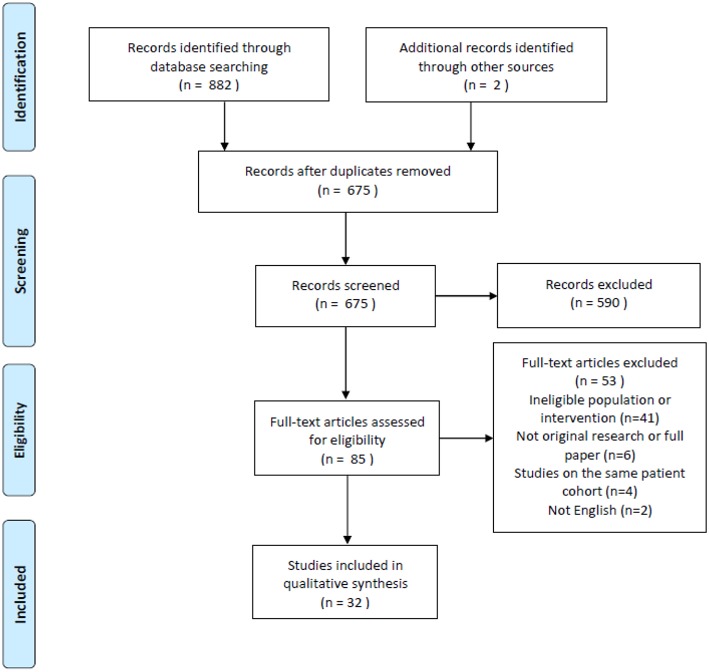
PRISMA flow chart of search results (19).

There were 882 search results with a total of 675 individual studies identified after duplicates were removed. Two studies were identified through the reference lists of relevant studies. Thirty-two studies were included after the screening process. The search process is illustrated in the PRISMA flow chart (Figure 1).

### Study Characteristics

Thirty-two studies were included after the screening process. Studies were published between 1987 and 2016. Study types include 12 case controls, 19 case series/reports and 1 cohort retrospective study.

**Figure 2:**
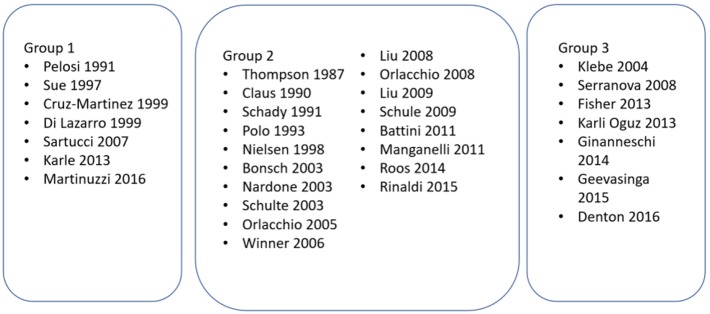
Studies grouped according to the aim/objective of the study. Group 1—Studies investigating the role of MEPs in HSP; Group 2—Studies where MEPs were performed to characterize the phenotype of HSP; Group 3—Studies where MEPs were performed as an adjunct to another intervention or investigation in HSP.

Studies were grouped according to their primary aims (see Figure 2).

#### Patient Demographics

Patient sample sizes ranged between 1 and 128 patients in each study. The mean number of patients who underwent MEPs per study was 14.88. 31/32 (97%) studies included fewer than 50 patients, 16/32 (48%) studies included 10 or fewer patients. Overall, there were a total of 476 patients with HSP who underwent MEPs in the studies reviewed.

Information on patient genotypes was included for 20/32 (63%) studies. 16/32 (50%) studies included patients with a single genotype, most commonly SPG4. 10/32 (31%) studies did not specify the patients' genotypes or only included patients with unknown genotypes. Six (19%) studies included patients with mixed genotypes and unknown genotypes. Several studies were performed before genetic testing was available, and the inheritance pattern was determined when possible.

The proportion of patients with pure and complicated phenotypes was described in 24/32 studies. Overall, 234 (76%) patients with pure HSP and 73 (24%) patients with complicated HSP were studied. Of the 23 studies that commented on the presence of peripheral neuropathy, either on examination or nerve conduction studies, there was a total of 130 out of 355 (37%) patients who had peripheral neuropathy.

The ratio of male:female patients was available for 21/32 studies, the overall ratio calculated from the 21 studies was 1.3. Mean age was available for 23/32 papers with an overall mean age of 40.93 years at the time of the study. Additionally, some papers included age at onset and disease duration. Thirteen out of 32 studies included patients from the same family.

#### Neurophysiological Techniques

Information on the type of stimulator used, type of coil used, stimulus intensity, number of stimuli administered, and muscles studied are provided in Table 1. Three studies used transcranial electrical stimulation whereas the remainder used transcranial magnetic stimulation (20–22).

**Table 1:** Details of neurophysiological techniques used in MEP studies.

**Details of Neurophysiological techniques**	**Number of studies with available information**	**Details**
Stimulator used	18/32	Dantec Maglite, MagStim 200, Twin top, Digitimer, interconnected electrodes
Coil used	17/32	Circular, double cone/parabolic/figure of 8
Stimulus intensity to elicit MEP	17/32	Most common 120% resting motor threshold but wide range used
Number of stimuli	11/32	Range from 3 stimuli to “at least 20”
Muscles studied	27/32	Most common tibialis anterior (16/26), abductor pollicis brevis (9/26), first dorsal interosseus (8/26), adductor digiti minimi (7/26), abductor hallucis (5/26)

CMCT calculation method was specified for 24/32 papers; 11 papers used the F-wave method, 11 papers used the spinal stimulation method and 2 studies used both methods (23, 24). Two studies used the F-wave method, however detailed a different peripheral motor conduction time calculation formula to previously published guidelines (13, 16, 25).

#### Clinical Rating Scales

Clinical rating scales were used in 15/32 studies with the most common being the modified Ashworth scale (5 studies) (23, 26–29), SPRS (4 studies) (13, 28, 30, 31) and MRC (4 studies) (27–30). Other rating scales used included functional grading scales (29, 32), disability staging scores (33–35), Functional Independence Measure (28), Barthel index (26), Behan-Maia modified scale (29), Abbreviated Mental Test Score (26), revised Wechsler Adult Intelligence Scale (28), Esame Neuropsicologico Breve 2 (28), Cambridge Cognition Examination (36, 37), and 6 min walk test (28).

Gait analysis was performed in one study investigating gait patterns in HSP (27).

### MEP Results

#### Central Motor Conduction Time

Overall, lower limb CMCT was more likely to be abnormal in patients with HSP compared to upper limb CMCT. Most (96%) studies that investigated lower limb CMCT reported abnormalities, with LL CMCT abnormalities in 308/393 (78%) patients with HSP that were studied (excluding 4 studies with unspecified UL/LL CMCT).

Fifty nine percent of studies that investigated upper limb CMCT reported abnormalities, with 93/282 HSP patients studied showing UL CMCT abnormalities (see Table 4.1 in Appendix 4).

**Chart 1:**
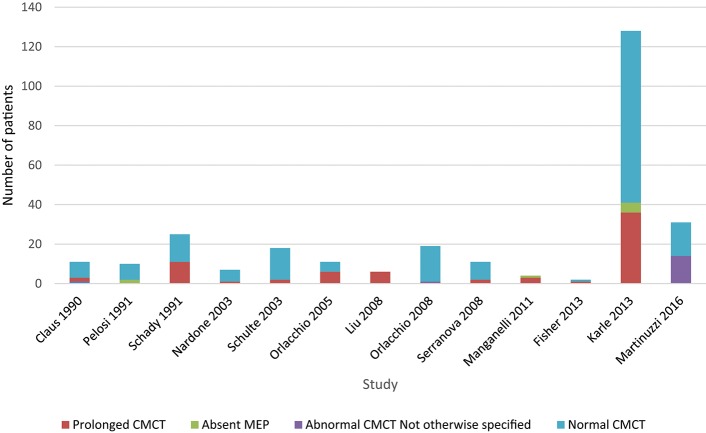
Studies of upper limb MEPs. Data available in Table 4.1, Appendix 4.

Upper limb CMCT results were reported for 22/32 (67%) studies and not studied in 7 papers. UL CMCT was found to be normal in all patients studied in 9/22 (41%) papers and abnormal in 13/22 (59%) studies (Chart 1, Tables 4.1, 4.2 in Appendix 4). In the 9 studies that reported normal UL CMCT in all patients, 5 studies were in exclusively SPG4 patient cohorts (23, 25, 30, 31, 34), 3 in HSP patient cohorts with unknown genotype (21, 38) and 1 in a patient with SPG31 (39) HSP. Overall, 93/359 (26%) patients studied had abnormal UL CMCT.

Sensitivity of upper limb CMCT abnormalities is 0.3.

**Chart 2:**
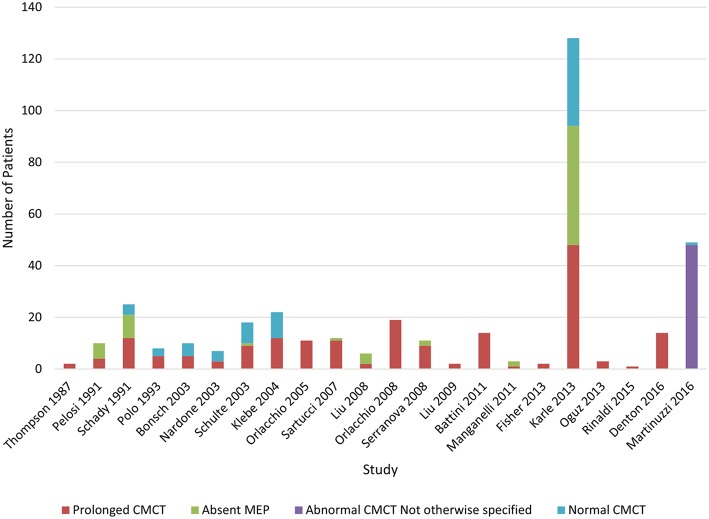
Studies of lower limb MEPs. Data available in Table 4.3, Appendix 4.

Lower limb CMCT results were reported for 26/32 (81%) studies and not studied in 3 papers. Only 1 study of 16 patients from 4 families with chromosome 2p linked HSP (SPG4) reported LL CMCT within the normal range for all patients although there was a tendency for delay in the lower limbs (34). LL CMCT was prolonged or absent for all patients in 8/26 studies and in some patients for 15/26 studies (Chart 2, Table 4.3 in Appendix 4). Overall, 308/393 (78%) patients studied had abnormal LL CMCT.

Sensitivity of lower limb CMCT abnormalities to diagnose HSP is 0.8, allowing for bias of only studying affected patients. This is the same as the result reached by Di Lazzaro et al. (24).

Four studies that did not distinguish between UL and LL CMCT all showed varying abnormalities in CMCT (Table 4.4 in Appendix 4).

**Chart 3:**
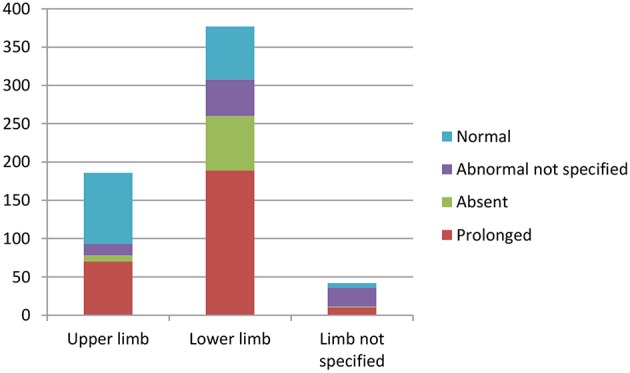
Summation of studies demonstrating number of patients with abnormal vs. normal central motor conduction times. Data available in Tables 4.1–4.4 in Appendix 4.

Chart 3 illustrates the summative CMCT results across all studies reviewed.

#### Amplitude

MEP amplitude was reported for UL in 8 papers, with lower amplitudes found in 5/8 studies and normal amplitudes in 3/8 studies (22, 25, 29, 30, 35, 40–42). LL MEP amplitude was studied in 12 papers with absent or reduced LL MEP amplitudes reported for some or all patients in all 12 studies (13, 20, 22, 23, 27–29, 33, 35, 40, 43, 44).

#### Resting Motor Threshold (RMT)

Resting motor threshold was reported in 7 studies. LL RMT was reported in 5 studies and found to be increased in all. UL RMT was reported in 4 studies and was noted to be normal in all.

#### Correlation of CMCT With Other Variables

Correlation between CMCT and other variables were investigated in 10/32 studies. In one paper, upper and lower limb CMCT was found to significantly correlate with SPRS scores (*r* = 0.176 and *r* = 0.234) and the spastic subscore (*r* = 0.241 and *r* = 0.300) whilst LL CMCT correlated with disease duration (*r* = 0.231) (13). Another study that included only 2 patients found that the more clinically severe patient had a prolonged CMCT (41). A further study found that tibialis anterior derived LL CMCT correlated weakly with disability but only in the early onset (<20 years of age) HSP subgroup (32).

Eight studies found no correlation between CMCT and other variables including disease severity, clinical signs, disease duration, age, age of onset, gait abnormalities, modified Ashworth score, and gender (23, 27–29, 32, 34, 35, 45).

#### Other Results

Cortical silent period (CSP) was measured using transcranial magnetic stimulation (TMS) in four studies, three of which showed no difference in HSP patients compared to controls (25, 30, 45). One study showed significantly reduced CSP for tibialis anterior, and this correlated with spasticity as measured by the Modified Ashworth Score (23).

Short-interval intracortical inhibition (SICI) was measured in three studies. One showed no difference in HSP patients compared to controls (30), another showed reduced SICI in SPG4 patients but not SPG7 (45) and the last was a case report of a SPG11 patient that demonstrated delayed transcallosal inhibition (44).

Two studies investigating MEP recruitment curves showed no difference in HSP patients compared to controls (25, 31). One study of TMS modulation of the soleus H reflex showed abnormal modulation in a pure HSP cohort (29).

One study not included in our analysis evaluated CMCT to the external anal sphincter in the SPG4 cohort described in Nielsen et al. (34). This demonstrated that a prolonged CMCT to the external anal sphincter correlated with lower urinary tract symptoms in this SPG4 cohort. Subjects without symptoms had similar CMCT compared to controls (46).

### Results by Genotype

#### SPG4

Thirteen papers included patients with *SPAST* (SPG4) mutations. Two studied just lower limb MEPs, two studied just upper limb, nine studied both. Nine studies provided an overall result for the whole SPG4 subgroup whilst the rest (four) provided individual patient results (at least in graph format). Eight included quantitative data on CMCTs (including graphed data) whilst the rest reported either “normal” or “prolonged” CMCT.

Of the 13 UL studies, 9 reported no difference in the CMCT compared to controls. A minority of abnormal cases were reported in the 4 other studies. Overall, 10/149 patients were reported to have abnormal UL CMCT.

Of the 12 LL studies, all reported prolonged CMCT in all or at least some of the patients studied. For the papers which reported individual results, the proportion of abnormality ranged from 10 to 100%. Although we cannot summate the results due to methodological differences and methods of reporting results, ~60–70% patients demonstrated abnormal results.

Notably, large variation was seen, despite mutations in the same gene. Bonsch et al. demonstrated abnormality in a family with an in-frame deletion of exons 13 to 16, but not in a family with a single base pair deletion resulting in a premature stop codon in exon 9 (25). Orlacchio et al. found significantly prolonged CMCTs in the males of a family with the same mutation, whereas the females had normal CMCTs (36). Karle et al. noted that mutation type influenced CMCT findings: SPG4 missense mutations were associated with shorter CMCT compared to patients with SPG4 splice site mutations, premature stop codon or in-frame deletions (13).

Three studies compared SPG4 patients with other genotypes. Schulte and colleagues compared SPG4 and non-SPG4 patients, finding MEP abnormalities were milder for SPG4 patients despite no clinical differences (47). Karle et al. found that SPG4 patients were more likely to have a “pure” HSP phenotype compared to non-SPG4 patients (60% vs. 36%) (13). In the cohort of Karle et al., UL and LL CMCTs were normal in most SPG4 patients but significantly longer in non-SPG4 HSP patients. Nardone et al. compared SPG4 and SPG7 and found no significant difference between the two patient groups aside from reduced short interval ICI in chromosome 2p linked HSP compared to normal ICI in chromosome 16q linked HSP (45).

#### Other Genotypes

Martinuzzi et al. performed a battery of clinical and neurophysiological tests (including MEPs) in a large cohort of genotypically characterized HSP patients (28). It was not specified how many of each HSP genotype underwent MEPs. Though the authors noted some correlations between SPRS and disease duration for some genotypes (SPG5, 7, 10), no correlation was found with MEPs. The authors did not draw any conclusions regarding an association of CMCT to any single HSP genotype.

Regarding other genotypes, it is hard to draw conclusions due to small numbers and incomplete reporting of results. Though most data were only qualitative, there were several mentions of *severely* prolonged CMCT in some cases of non-SPG4 patients which may imply a demyelinating rather than axonal pathophysiology (13, 47). SPG5/5A, 6, 7 and 11 tended to be prolonged, though not in *all* cases (13, 35, 37, 40, 47, 48).

### Methodological Quality Assessment: Risk of Bias

The studies were methodologically evaluated by two reviewers and the full results can be found in Appendix 3. Validated tools for clinical series and case control studies were used. Using the NIH Study Quality Assessment Tool, case series studies were scored between 2 and 8 out of 8 criteria (17). For case-control studies, the two criteria relating to a study “exposure” were not appropriate for HSP and were discounted. We deemed that studies scored between 3 and 6 out of a possible 9. It was found that the aims and objectives of the studies were adequately published in most cases, though some papers were only used to report a clinical phenotype, rather than answer a more specific question related to MEPs (see Figure 2). The study methodology was reported variably, and poorly in some cases. Though some papers used concurrent controls, others relied upon laboratory values or historical controls. Only some papers mentioned efforts to match the controls for age, sex, and other variables (24–26, 30, 46). The nature of the cases meant that risk of bias was high, and selection of patients from a proband may have resulted in bias. Furthermore, no study mentioned that the MEP assessment was blinded to the diagnosis, and prior knowledge of the diagnosis may have introduced bias.

Similarly, the results of the studies were variably reported. Some papers only mentioned prolongation of CMCTs in a single sentence, whereas others provided tables and graphs of individual CMCT results, as well as their comparative control results. 15/32 studies reported individual results (some on a graph), 15/32 reported results for the group, and 1 study reported both. 17/32 studies provided quantitative results, whereas 16/32 were only qualitative (e.g., “prolonged” CMCT). This variability in reporting of results prevented amalgamation of the data, precluding us from performing a meta-analysis.

The TMS methodology checklist demonstrated significant variability between studies (18). Studies scored between 0 and 22 of the 24 criteria. Studies focused on MEPs often outlined their methodology in detail (including muscles studied, stimulator, coil, and stimuli given). Other studies which were only using MEPs as an ancillary test, and those concentrating on other aspects of HSP, did not include sufficient detail (28, 33, 35–37, 39, 40, 44, 47–50). Only a few studies mentioned the presence of possible confounders such as medications and other medical conditions (29, 32, 42, 44).

## Discussion

This systematic review showed that prolonged or absent lower limb CMCT is a potential diagnostic biomarker for HSP. However, only one study showed a weak correlation between upper and lower limb CMCT and clinical disease severity. Hence, the utility of CMCT as a prognostic biomarker in HSP remains uncertain.

### CMCT as a Diagnostic Biomarker for HSP

Most (96%) of studies reported prolongation of CMCT or absence of MEPs in the lower limbs with 78% of patients studied demonstrating lower limb CMCT abnormalities, whilst only 59% of studies reported abnormalities in the upper limbs. The gold standard for the diagnosis of HSP was considered to be appropriate findings on clinical exam, appropriate exclusion of other disorders, and genetic testing. Compared to this standard, this review found that prolongation of lower limb CMCT had a sensitivity of 0.8.

The underlying disease pathophysiology of HSP is length-dependent axonal degeneration (2). CMCT measured from MEPs are thought to reflect neuronal integrity and are therefore a potential surrogate marker of disease severity. In this systematic review, CMCT was more likely to be abnormal in the lower limb (78%) vs. the upper limb (26%) consistent with more neuronal damage in the longer motor tracts to the lower limbs. This was also seen when HSP was compared to other motor neuron diseases such as hereditary motor and sensory neuropathy (HMSN) types 1 and 2, amyotrophic lateral sclerosis (ALS) and primary lateral sclerosis (PLS), where CMCT to the upper limbs were more likely to be normal in HSP compared to these other conditions (30, 41, 42). This finding was also confirmed in a study using the triple stimulation technique, thought to be the most accurate neurophysiological measure of upper motor neuron integrity, which found normal central motor conduction to the upper limbs in 15 patients with pure HSP (13 had SPG4 HSP) (51).

However, there was a significant proportion of patients with normal CMCT to the upper and lower limbs despite severe signs of spasticity. In fact, most studies did not find any correlation of CMCT abnormalities with disease severity, disease duration or age of onset (23, 27–29, 32, 34, 35, 45). There are several possible explanations for this finding. One could be that although not significantly prolonged compared to controls, the CMCT of affected patients may be prolonged compared to their baseline before developing symptoms. Two studies showed borderline or mild prolongation in CMCT in patients with HSP compared to controls although these were reported as normal as the prolongation was not significant (30, 34). A longitudinal study of MEPs in patients with HSP, ideally from presymptomatic to symptomatic stage might shed light on the theory that increasing CMCT occurs in the presymptomatic stage and may not change once the patients is symptomatic, indicative of a ceiling effect of the test (see later).

Another explanation is that although symptoms are associated with neuronal damage, there may be enough residual nerve fibers that are intact to conduct the action potentials induced with transcranial magnetic stimulation or transcranial electrical stimulation. There are limited neuropathological studies in HSP as it typically does not reduce life span. One study looked at cervical and lumbar spinal cord sections from two patients with HSP and found axonal swellings in the long descending corticospinal (CST) axons, more prevalent in the dorsal column (52). Another looked at post-mortem tissue from six HSP patients and showed a significant reduction in axonal density and number of axons of CST and sensory tracts compared to controls (53). The latter study demonstrated a reduction of both larger and smaller diameter fibers equally within the CST, however the integrity of the remaining neurons was not described and these could be enough to maintain central motor conduction without sufficiently demonstrating slow conduction in a grouped axon test.

It is also likely that clinical motor impairment in HSP is due to more global cerebral impairment than just the motor tracts. A study using magnetoencephalography to assess the connectivity of brain networks found changes that suggested global network rearrangement due to changes beyond motor impairment (54). One study looking at 118 patients with SPG4 HSP found 10% had psychiatric comorbidities and 3.5% had memory impairment, possible reflecting more global cerebral impairment (55).

There are several limitations of MEPs as a clinical biomarker. A recent paper investigating the use of MEP measures in movement disorders, specifically Parkinson's disease, dystonia, Tourette syndrome, Huntington Disease and essential tremor, found discrepancies between studies reporting “canonical” MEP findings for these conditions (56). Similar to our findings, the authors found inconsistencies in methodology, diagnostic criteria for inclusion, study participants' disease stages, and small sample sizes contributed to weaker evidence for the use of MEPs for diagnosis and differential diagnosis in movement disorders. See below for limitations of MEPs as a biomarker in HSP.

Limitations of MEPs as a biomarker in HSP

- Limitations of MEPs
  ∘ Low to moderate reliability of some MEP measures, e.g., MEP recruitment, cortical silence period, intracortical facilitation (56)
  ∘ Peripheral neuropathy causing CMCT prolongation due to slowing in the cauda equina (16)
  ∘ Cognitive impairment may affect patient's ability to actively participate

- HSP-related factors
  ∘ Slow progression of disease
  ∘ Low prevalence
  ∘ Clinically and genetically heterogenous
  ∘ No previously known biomarker

- Limitations of reviewed studies
  ∘ Variability in study methodology
  ∘ Small sample size of patient cohorts studied
  ∘ Clinically and genetically heterogeneous patient samples

In studies where HSP was investigated along with other neurodegenerative conditions, CMCT changes in HSP were found to be milder than in multiple sclerosis, myelopathy, stroke and motor neuron disease (20, 21, 24). Therefore, characteristic changes of prolonged or absent LL CMCT with normal or mildly prolonged UL CMCT are more likely to suggest a diagnosis of HSP, whilst patients with grossly abnormal UL and LL CMCT are more likely to be seen in other types of motor neuron disease. However, it is important to note that these changes are not specific to HSP and can be seen in other motor neuron conditions (20).

In summary, although useful in diagnosis of HSP, CMCT is best used to confirm the clinical examination findings or the results of genetic testing.

### CMCT as a Measure of Treatment Response for HSP

Only 30% of studies that investigated correlation between CMCT and other clinical variables reported a mild correlation with disease severity. One study only included two patients whilst the other only showed a correlation in patients with disease onset before 20 years old (32, 41). It is difficult to draw conclusions from these results, but the current evidence does not support a strong correlation with disease severity.

MEPs have not been studied longitudinally in HSP and therefore, the ability to assess changes in disease severity over time remains to be established. It is possible that individual patients may show changes from their baseline MEPs over time although some may not be in the abnormal range, whilst in others, their MEPs may become absent before their CMCT is in the abnormal range. Patients may exhibit a “ceiling” effect where after a certain degree of motor neuron damage, CMCT measurements remain unchanged or absent. Studies investigating the change in MEP parameters longitudinally in HSP patients across different disease stages will help clarify the ability of MEPs to reflect disease progression.

None of the studies reviewed included presymptomatic patients with confirmed genetic diagnoses. MEP studies in this specific group of patients will help shed light on the utility of CMCT to predict future motor impairment. MEPs may be more sensitive to changes early in the disease process, before significant motor neuron damage limits variation in MEP measures.

Overall, longitudinal studies of MEPs in patients with HSP, including presymptomatic and various stages of the disease, are required to establish the utility of CMCT as a prognostic biomarker. Similarly, information from such studies will reveal the natural history of upper motor neuron damage in HSP.

### Impact of HSP Genotype on MEP Findings

Fifty two percent of studies reviewed included patients from a single genotype, however, most of these were case reports or case series. The largest studies of MEP in HSP included a heterogeneous cohort of patients with multiple genotypes and unknown genotypes (13, 23). In addition, these studies did not clearly delineate MEP changes according to each genotype, with the exception of the study by Karle and colleagues which performed subgroup analysis on SPG4 patients as well as providing values for other genotypes tested (13). Therefore, it is difficult to draw conclusions on MEP findings specific to each genotype although some patterns do emerge.

The largest cohorts that studied genotypically confirmed SPG4 patients noted that SPG4 patients had normal or only mildly prolonged MEPs (13, 34). These papers hypothesized that a very prolonged CMCT makes an SPG4 genotype unlikely, and may indicate SPG5, 6, 7, or 11. A study by Schulte et al. suggested that MEPs were a useful way of differentiating SPG4 from non-SPG4 HSP (47). Orlacchio et al. provides a caveat to this: all males in the SPG4 family studied had very prolonged CMCTs, whereas the women did not (36). This finding has not been replicated and the mechanism underlying this observation is uncertain.

For the other genotypes, the study numbers were too small to draw any firm conclusions. Though a significantly prolonged CMCT may point to certain HSP genotypes, patients will still need to undergo genetic testing to identify the mutation.

### Future Recommendations

Further studies of MEPs in HSP with standardized methodology, strict inclusion criteria, adequate sample sizes and standardized assessment of clinical disease severity will clarify the role of MEP measures in HSP diagnosis and monitoring of disease severity. Our systematic review does not strongly support the use of MEPs as a sole biomarker in HSP although it remains useful when combined with other biomarkers, including clinical rating scales and diffusion tensor imaging.

There remains a need for a biomarker for use in future HSP clinical therapeutic trials. An ideal biomarker will be able to measure small changes in disease severity seen in HSP to account for the relatively short duration of clinical trials (1–2 years). Future studies of other potential biomarkers, such as neuroimaging and biological fluid-based biomarkers, are needed.

## Conclusion

In summary, MEPs are not superior to clinical examination and genetic testing for diagnosis for HSP. However, MEP findings may help confirm a clinical diagnosis in suspected patients. Prospective longitudinal studies in presymptomatic and symptomatic patients with known genotypes are needed to clarify the utility of MEPs as a prognostic biomarker for HSP. Overall, this systematic review has revealed variation in MEP findings in patients in HSP with the most consistent finding being prolonged lower limb CMCT over upper limb abnormalities. In fact, the presence of greater lower limb involvement may be more likely to signify the presence of HSP when compared to other upper motor neuron disorders. Study quality assessment has shown inconsistencies in study methodology and reporting of results, perhaps contributing to the variation in results and preventing meta-analysis of available data. Current studies are insufficient to establish the validity of MEPs as a prognostic marker or a measure of disease severity as most were not designed for this purpose. Nevertheless, no clear correlation was found between MEP abnormalities and disease severity or duration, but there was some tendency for certain subtypes (e.g., SPG4) to be less affected. Future longitudinal studies in HSP patients with known genotypes, investigating the correlation of MEP parameters with standardized measures of clinical disease severity will help clarify the use of MEPs as a surrogate marker for disease severity for use in future clinical drug trials.

## Data Availability

The raw data supporting the conclusions of this manuscript will be made available by the authors, without undue reservation, to any qualified researcher.

## Author Contributions

S-FS and KK conceived the study. S-FS performed the database search, initial screening, and developed the data extraction form. S-FS and RC extracted and analyzed the data and drafted the manuscript. KK, KN, and CS provided advice, critically reviewed the manuscript, and approved the manuscript for publication.

### Conflict of Interest Statement

The authors declare that the research was conducted in the absence of any commercial or financial relationships that could be construed as a potential conflict of interest.
