# Supplementary material for: Motor Evoked Potentials in Hereditary Spastic Paraplegia—A Systematic Review
Source: Front Neurol. 2019 Sep 18;10:967. doi: 10.3389/fneur.2019.00967 (PMC6759520; doi:10.3389/fneur.2019.00967)
Supplement: Supplementary file 1 [file Data_Sheet_1.docx]

Supplementary Material

# Appendix 1 – Search Strategies

Searches performed on 13 March 2019

## Database: PubMed

Search strategy:

((((hereditary spastic paraplegia) OR spastic paraparesis) OR HSP)) AND ((((((motor evoked potential) OR MEP) OR transcranial magnetic stimulation) OR TMS) OR central motor conduction time) OR CMCT)

Results: 121

## Database: Scopus

ALL ( ( "hereditary spastic parap*"  OR  "spastic parap*"  OR  "HSP" )  AND  ( "motor evoked potential*"  OR  "MEP"  OR  "transcranial magnetic stimulation"  OR  "TMS"  OR  "central motor conduction*"  OR  "CMCT" ) )

Limited to journals, English, article

Results: 547

## Database: Embase

Search Strategy:

[(“hereditary motor sensory neuropathy/” OR “spastic paraplegia/” OR “spastic paraparesis.mp.” OR “spastic para*.mp.” OR “HSP.mp.”) AND (“motor evoked potential/” OR “motor evoked potential*.mp.” OR “MEP.mp.” OR “transcranial magnetic stimulation/” OR “transcranial magnetic stimulation*.mp.” OR “TMS.mp.” OR “central motor conduction time*.mp.” OR “CMCT.mp.”)]

Limit to (human and english language)

Results: 146

## Database: Ovid MEDLINE(R)

Search Strategy:

[(“Spastic Paraplegia, Hereditary/” OR “Paraparesis, Spastic/” OR “spastic paraparesis.mp.” OR “spastic para*.mp.” OR “HSP.mp.”) AND (“Evoked Potentials, Motor/” OR “motor evoked potential*.mp.” OR “MEP.mp.” OR “Transcranial Magnetic Stimulation/” OR “transcranial magnetic stimulation*.mp.” OR “TMS.mp.” OR “central motor conduction time*.mp.” OR “CMCT.mp.”)]

Limit to (english language and humans)

Results: 68

# Appendix 2 - Data Collection Form

## Study Characteristics

- Study ID
- Study Author
- Year of publication
- Reviewer
- Study type
- Patient number
- Genotype known
- Genotype unknown
- Males
- Females
- Mean age
- Age SD or Range
- Number of controls
- Other patient details
- Clinical scales used

## Neurophysiological Techniques

- Stimulator
- Coil
- Stimulation intensity
- Number of stimuli
- Muscles studied
- Other parameters studied
- CMCT calculation method

## Results

- Results - UL CMCT
- Results - LL CMCT
- Results – Amplitude
- Results – resting MT
- Results - Correlation
- Results – Other

# Appendix 3

3.1 NIH Study Quality Assessment Tool (* items not included as not relevant to current study)

### Quality Assessment of Case-Control Studies

1. Was the research question or objective in this paper clearly stated and appropriate?
2. Was the study population clearly specified and defined?
3. Did the authors include a sample size justification?
4. Were controls selected or recruited from the same or similar population that gave rise to the cases (including the same timeframe)?
5. Were the, inclusion and exclusion criteria, algorithms or processes used to identify or select cases and controls valid, reliable, and implemented consistently across all study participants?
6. Were the cases clearly defined and differentiated from controls?
7. If less than 100 percent of eligible cases and/or controls were selected for the study, were the cases and/or controls randomly selected from those eligible?*
8. Was there use of concurrent controls?
9. Were the investigators able to confirm that the exposure/risk occurred prior to the development of the condition or event that defined a participant as a case?*
10. Were the measures of exposure/risk clearly defined, valid, reliable, and implemented consistently (including the same time period) across all study participants?*
11. Were the assessors of exposure/risk blinded to the case or control status of participants?
12. Were key potential confounding variables measured and adjusted statistically in the analyses? If matching was used, did the investigators account for matching during study analysis definitions?

| Author | Year | 1 | 2 | 3 | 4 | 5 | 6 | 8 | 11 | 12 | TOTAL (9) |
| --- | --- | --- | --- | --- | --- | --- | --- | --- | --- | --- | --- |
| Thompson | 1987 | 1 | 1 | 0 | 0 | 1 | 1 | 1 | 0 | 0 | 5 |
| Claus | 1990 | 1 | 1 | 0 | 0 | 1 | 1 | 0 | 0 | 0 | 4 |
| Schady | 1991 | 1 | 1 | 0 | 1 | 1 | 1 | 1 | 0 | 0 | 6 |
| Sue | 1997 | 0 | 1 | 0 | 0 | 0 | 1 | 1 | 0 | 0 | 3 |
| Di Lazarro | 1999 | 1 | 1 | 0 | 1 | 1 | 1 | unclear | 0 | 1 | 6 |
| Bonsch | 2003 | 1 | 1 | 0 | 1 | 1 | 1 | unclear | 0 | 1 | 6 |
| Sartucci | 2007 | 1 | 1 | 0 | 1 | 1 | 1 | 1 | 0 | 0 | 6 |
| Fisher | 2013 | 1 | 1 | 0 | 1 | 1 | 1 | unclear | 0 | 0 | 5 |
| Oguz | 2013 | 1 | 1 | 0 | 1 | 1 | 1 | 1 | 0 | 0 | 6 |
| Giananneschi | 2014 | 1 | 1 | 0 | 1 | 1 | 1 | unclear | 0 | 1 | 6 |
| Geevasinga | 2015 | 1 | 1 | 0 | 0 | 1 | 1 | unclear | 0 | 1 | 5 |
| Denton | 2016 | 1 | 1 | 0 | 0 | 1 | 1 | 1 | 0 | 1 | 6 |
| Martinuzzi | 2016 | 1 | 1 | 0 | 0 | 1 | 1 | 0 | 0 | 0 | 4 |

Table 3.1.1 Scores for quality assessment of case-control studies

### Quality Assessment Tool for Case Series Studies

1. Was the study question or objective clearly stated?
2. Was the study population clearly and fully described, including a case definition?
3. Were the cases consecutive?
4. Were the subjects comparable?
5. Was the intervention clearly described?
6. Were the outcome measures clearly defined, valid, reliable, and implemented consistently across all study participants?
7. Was the length of follow-up adequate?*
8. Were the statistical methods well-described?
9. Were the results well-described?

| Author | Year | 1 | 2 | 3 | 4 | 5 | 6 | 8 | 9 | TOTAL (8) |
| --- | --- | --- | --- | --- | --- | --- | --- | --- | --- | --- |
| Pelosi | 1991 | 1 | 1 | 0 | 1 | 1 | 1 | 0 | 1 | 6 |
| Polo | 1993 | 1 | 1 | 0 | 1 | 1 | 1 | 0 | 1 | 6 |
| Nielsen | 1998 | 1 | 1 | 0 | 1 | 1 | 1 | 1 | 1 | 7 |
| Cruz | 1999 | 0 | 1 | 1 | 1 | 1 | 1 | 0 | 1 | 6 |
| Nardone | 2003 | 1 | 1 | 0 | 1 | 1 | 1 | 1 | 1 | 7 |
| Schulte | 2003 | 1 | 1 | 0 | 1 | 0 | 0 | 0 | 0 | 3 |
| Klebe | 2004 | 1 | 1 | 0 | 1 | 1 | 1 | 1 | 1 | 7 |
| Orlacchio | 2005 | 1 | 1 | 1 | 1 | 0 | 0 | 0 | 1 | 5 |
| Winner | 2006 | 1 | 1 | 0 | 0 | 0 | 0 | 0 | 0 | 2 |
| Liu | 2008 | 1 | 1 | 1 | 1 | 0 | 1 | 0 | 0 | 5 |
| Serranova | 2008 | 1 | 1 | 1 | 1 | 1 | 1 | 1 | 1 | 8 |
| Orlacchio | 2008 | 0 | 0 | 0 | 0 | 0 | 0 | 0 | 0 | 6 |
| Liu | 2009 | 1 | 1 | 1 | 1 | 0 | 1 | 0 | 1 | 6 |
| Schule | 2009 | 1 | 1 | 1 | 1 | 0 | 0 | 0 | 0 | 4 |
| Battini | 2011 | 1 | 1 | 0 | 1 | 0 | 1 | 1 | 1 | 6 |
| Manganelli | 2011 | 1 | 1 | 1 | 1 | 1 | 1 | 1 | 1 | 8 |
| Karle | 2013 | 1 | 1 | 0 | 0 | 1 | 0 | 1 | 1 | 5 |
| Roos | 2014 | 1 | 1 | 1 | 1 | 0 | 0 | 0 | 0 | 4 |
| Rinaldi | 2015 | 1 | 1 | 0 | 0 | 0 | 0 | 0 | 0 | 2 |

Table 3.1.2 Scores for quality assessment of case series studies

## Chipchase TMS checklist (* items not included as not relevant to current study)

### Were the following participant factors reported/controlled?

1. Age of subjects
2. Gender of subjects
3. Handedness of subjects
4. Subjects prescribed medication
5. Use of CNS active drugs
6. Presence of neurological/psychiatric disorders when studying healthy subjects *
7. Any medical conditions
8. History of specific repetitive motor activity *

### Were the following methodological factors reported/controlled?

1. Position and contact of EMG electrodes
2. Amount of relaxation/contraction of target muscles
3. Prior motor activity of the muscle to be tested
4. Level of relaxation of muscles other than those being tested
5. Coil type (size and geometry)
6. Coil orientation
7. Direction of induced current in the brain
8. Coil location and stability (with or without a neuronavigation system)
9. Type of stimulator used (e.g. brand)
10. Stimulation intensity
11. Pulse shape (monophasic or biphasic)
12. Determination of optimal hotspot
13. The time between MEP trials
14. Time between days of testing *
15. Subject attention (level of arousal) during testing
16. Method for determining threshold (active/resting)
17. Number of MEP measures made
18. Paired pulse only: Intensity of test pulse *
19. Paired pulse only: Intensity of conditioning pulse *
20. Paired pulse only: Inter-stimulus interval *

### Were the following analytical factors reported/controlled?

1. Method for determining MEP size during analysis
2. Size of unconditioned MEP

| **Author** | **Year** | **1** | **2** | **3** | **4** | **5** | **7** | **9** | **10** | **11** | **12** | **13** | **14** |
| --- | --- | --- | --- | --- | --- | --- | --- | --- | --- | --- | --- | --- | --- |
| Thompson | 1987 | 1 | 1 | 0 | 0 | 0 | 0 | 1 | 1 | 0 | 1 | 1 | 0 |
| Claus | 1990 | 1 | 1 | 0 | 0 | 0 | 1 | 1 | 1 | 0 | 0 | 1 | 1 |
| Pelosi | 1991 | 1 | 1 | 0 | 0 | 0 | 0 | 1 | 1 | 0 | 0 | 1 | 1 |
| Schady | 1991 | 1 | 1 | 0 | 0 | 0 | 1 | 1 | 1 | 0 | 1 | 1 | 1 |
| Polo | 1993 | 1 | 0 | 0 | 0 | 0 | 0 | 1 | 0 | 0 | 0 | 1 | 0 |
| Sue | 1997 | 1 | 1 | 0 | 0 | 0 | 0 | 1 | 1 | 0 | 1 | 1 | 0 |
| Nielsen | 1998 | 0 | 0 | 0 | 0 | 0 | 0 | 1 | 1 | 0 | 1 | 0 | 0 |
| Cruz | 1999 | 1 | 1 | 0 | 0 | 0 | 0 | 1 | 1 | 0 | 0 | 0 | 0 |
| Di Lazarro | 1999 | 0 | 0 | 0 | 0 | 0 | 0 | 1 | 1 | 0 | 0 | 1 | 1 |
| Bonsch | 2003 | 1 | 1 | 0 | 0 | 0 | 0 | 1 | 0 | 0 | 0 | 1 | 0 |
| Nardone | 2003 | 0 | 1 | 0 | 0 | 0 | 0 | 1 | 1 | 0 | 0 | 1 | 0 |
| Schulte | 2003 | 1 | 1 | 0 | 0 | 0 | 0 | 1 | 0 | 0 | 0 | 0 | 0 |
| Klebe | 2004 | 1 | 1 | 0 | 0 | 0 | 0 | 1 | 1 | 0 | 0 | 1 | 1 |
| Orlacchio | 2005 | 1 | 1 | 0 | 0 | 0 | 0 | 1 | 0 | 0 | 0 | 0 | 0 |
| Winner | 2006 | 1 | 1 | 0 | 1 | 0 | 0 | 0 | 0 | 0 | 0 | 0 | 0 |
| Sartucci | 2007 | 1 | 1 | 0 | 0 | 0 | 0 | 1 | 1 | 0 | 1 | 1 | 1 |
| Orlacchio | 2008 | 0 | 0 | 0 | 0 | 0 | 0 | 1 | 0 | 0 | 0 | 0 | 0 |
| Serranova | 2008 | 1 | 1 | 0 | 0 | 0 | 1 | 1 | 1 | 0 | 1 | 1 | 0 |
| Liu | 2008 | 1 | 0 | 0 | 0 | 0 | 0 | 1 | 0 | 0 | 0 | 0 | 0 |
| Liu | 2009 | 1 | 1 | 0 | 0 | 0 | 0 | 1 | 0 | 0 | 0 | 0 | 0 |
| Schule | 2009 | 0 | 0 | 0 | 0 | 0 | 0 | 0 | 0 | 0 | 0 | 0 | 0 |
| Battini | 2011 | 1 | 1 | 0 | 0 | 0 | 0 | 0 | 0 | 0 | 0 | 0 | 0 |
| Manganelli | 2011 | 1 | 1 | 0 | 0 | 0 | 0 | 1 | 0 | 0 | 0 | 0 | 0 |
| Fisher | 2013 | 1 | 1 | 0 | 0 | 0 | 0 | 1 | 1 | 0 | 0 | 1 | 1 |
| Karle | 2013 | 1 | 1 | 0 | 0 | 0 | 0 | 1 | 1 | 0 | 0 | 0 | 0 |
| Oguz | 2013 | 1 | 1 | 0 | 0 | 0 | 0 | 1 | 0 | 0 | 0 | 0 | 0 |
| Giananneschi | 2014 | 1 | 1 | 0 | 0 | 0 | 0 | 1 | 1 | 0 | 0 | 1 | 1 |
| Roos | 2014 | 1 | 1 | 0 | 0 | 0 | 0 | 0 | 0 | 0 | 0 | 0 | 0 |
| Geevasinga | 2015 | 1 | 1 | 0 | 0 | 0 | 0 | 1 | 1 | 0 | 0 | 1 | 0 |
| Rinaldi | 2015 | 1 | 1 | 0 | 0 | 0 | 0 | 0 | 0 | 0 | 0 | 0 | 0 |
| Denton | 2016 | 1 | 1 | 0 | 0 | 0 | 0 | 1 | 1 | 1 | 0 | 1 | 0 |
| Martinuzzi | 2016 | 0 | 0 | 0 | 0 | 0 | 0 | 0 | 0 | 0 | 0 | 0 | 0 |

| **Author** | **Year** | **15** | **16** | **17** | **18** | **19** | **20** | **21** | **23** | **24** | **25** | **29** | **30** | **TOTAL (24)** |
| --- | --- | --- | --- | --- | --- | --- | --- | --- | --- | --- | --- | --- | --- | --- |
| Thompson | 1987 | 0 | 1 | 1 | 1 | 1 | 0 | 0 | 0 | 0 | 0 | 1 | 1 | 12 |
| Claus | 1990 | 0 | 1 | 1 | 1 | 0 | 0 | 0 | 0 | 0 | 1 | 0 | 0 | 11 |
| Pelosi | 1991 | 1 | 1 | 1 | 1 | 0 | 1 | 0 | 0 | 0 | 0 | 1 | 1 | 13 |
| Schady | 1991 | 0 | 1 | 1 | 1 | 0 | 0 | 0 | 9 | 0 | 1 | 0 | 1 | 22 |
| Polo | 1993 | 0 | 0 | 1 | 1 | 0 | 0 | 0 | 0 | 0 | 1 | 0 | 0 | 6 |
| Sue | 1997 | 0 | 1 | 1 | 1 | 0 | 1 | 0 | 0 | 0 | 1 | 0 | 1 | 12 |
| Nielsen | 1998 | 0 | 1 | 0 | 1 | 0 | 1 | 0 | 0 | 0 | 0 | 0 | 0 | 6 |
| Cruz | 1999 | 0 | 1 | 1 | 1 | 0 | 0 | 0 | 0 | 0 | 1 | 0 | 0 | 8 |
| Di Lazarro | 1999 | 1 | 1 | 1 | 1 | 0 | 0 | 0 | 0 | 0 | 0 | 0 | 0 | 8 |
| Bonsch | 2003 | 0 | 0 | 1 | 1 | 0 | 0 | 0 | 0 | 0 | 1 | 1 | 0 | 8 |
| Nardone | 2003 | 0 | 0 | 1 | 1 | 0 | 0 | 1 | 1 | 1 | 1 | 1 | 0 | 11 |
| Schulte | 2003 | 0 | 0 | 0 | 0 | 0 | 0 | 0 | 0 | 0 | 0 | 0 | 0 | 3 |
| Klebe | 2004 | 0 | 0 | 1 | 1 | 0 | 0 | 0 | 0 | 0 | 1 | 0 | 0 | 9 |
| Orlacchio | 2005 | 0 | 0 | 0 | 0 | 0 | 0 | 0 | 0 | 0 | 0 | 0 | 0 | 3 |
| Winner | 2006 | 0 | 0 | 0 | 0 | 0 | 0 | 0 | 0 | 0 | 0 | 0 | 1 | 4 |
| Sartucci | 2007 | 1 | 1 | 1 | 1 | 1 | 1 | 0 | 0 | 1 | 1 | 1 | 1 | 17 |
| Orlacchio | 2008 | 0 | 0 | 0 | 0 | 0 | 0 | 0 | 0 | 0 | 0 | 0 | 0 | 1 |
| Serranova | 2008 | 0 | 0 | 1 | 1 | 0 | 0 | 0 | 0 | 1 | 0 | 1 | 1 | 12 |
| Liu | 2008 | 0 | 0 | 0 | 0 | 0 | 0 | 0 | 0 | 0 | 0 | 0 | 0 | 2 |
| Liu | 2009 | 0 | 0 | 0 | 0 | 0 | 0 | 0 | 0 | 0 | 0 | 0 | 0 | 3 |
| Schule | 2009 | 0 | 0 | 0 | 0 | 0 | 0 | 0 | 0 | 0 | 0 | 0 | 0 | 0 |
| Battini | 2011 | 0 | 0 | 0 | 0 | 0 | 0 | 0 | 0 | 0 | 0 | 0 | 0 | 2 |
| Manganelli | 2011 | 0 | 0 | 0 | 0 | 0 | 0 | 0 | 0 | 0 | 0 | 0 | 0 | 3 |
| Fisher | 2013 | 1 | 1 | 1 | 1 | 0 | 1 | 0 | 0 | 1 | 1 | 1 | 0 | 14 |
| Karle | 2013 | 0 | 0 | 0 | 1 | 0 | 0 | 0 | 0 | 1 | 0 | 0 | 0 | 6 |
| Oguz | 2013 | 0 | 0 | 0 | 0 | 0 | 0 | 0 | 0 | 0 | 0 | 0 | 0 | 3 |
| Giananneschi | 2014 | 1 | 1 | 1 | 1 | 1 | 1 | 0 | 0 | 1 | 1 | 1 | 1 | 16 |
| Roos | 2014 | 0 | 0 | 0 | 0 | 0 | 0 | 0 | 0 | 0 | 0 | 0 | 0 | 2 |
| Geevasinga | 2015 | 0 | 1 | 1 | 1 | 1 | 1 | 0 | 0 | 1 | 1 | 0 | 0 | 12 |
| Rinaldi | 2015 | 0 | 0 | 0 | 0 | 0 | 0 | 0 | 0 | 0 | 0 | 0 | 0 | 2 |
| Denton | 2016 | 1 | 0 | 1 | 1 | 0 | 0 | 0 | 0 | 1 | 1 | 0 | 0 | 11 |
| Martinuzzi | 2016 | 0 | 0 | 0 | 0 | 0 | 0 | 0 | 0 | 0 | 0 | 0 | 0 | 0 |

Table 3.2 Scores for Chipchase TMS checklist

# Appendix 4

## Studies with abnormal UL CMCT results

| **Study** | **Genotype** | **Results - prolonged** | **Results - absent** | **Results – total abnormal** |
| --- | --- | --- | --- | --- |
| Claus 1990 | Unknown, pure HSP | 2/10 (20%) |  | 2/10 (20%) |
| Pelosi 1991 | Unknown, pure HSP |  | 2/10 (20%) | 2/10 (20%) |
| Schady 1991 | Unknown, ADPSP | 11/25 |  | 11/25 (44%) |
| Nardone 2003 | SPG4, SPG7 | 1/7 (14%) SPG4 |  | 1/7 (14%) |
| Schulte 2003 | SPG4, Non-SPG4 | Prolonged in 2/10 (20%) Non-SPG4 patients  Normal in 8/8 SPG4 patients |  | 2/18 (11%) |
| Orlacchio 2005 | SPG4 | 6/11 (55%) |  | 6/11 (55%) |
| Liu 2008 | SPG6 | 6/6 (100%) |  | 6/6 (100%) |
| Orlacchio 2008 | SPG38 | NS | NS | 1/19 (5%) |
| Serranova 2008 | Pure HSP | 2/11 (18%) |  | 2/11 (18%) |
| Manganelli 2011 | SPG5 | 3/4 (75%) | 1/4 (25%) | 4/4 (100%) |
| Fisher 2013 | SPG31 | 1/2 (50%) |  | 1/2 (50%) |
| Karle 2013 | Mixed genotype | 36/128 (28%) | 5/128 (4%) | 41/128 (32%) |
| Martinuzzi 2016 | Mixed genotype | NS | NS | 14/31 (45%) |
| TOTAL |  |  |  | 93/282 (33%) |

Table 4.1 Studies with abnormal upper limb central motor conduction time. SPG31 (HSP-*REEP1*), SPG6 (HSP-*NIPA1*), SPG5 (HSP-*CYP7B1*), SPG4 (HSP-*SPAST*), SPG7 (HSP-*paraplegin*), SPG38 (HSP-*SPG38*).

## Studies with normal UL CMCT results

| **Study** | **Genotype** | **Number of patients** |
| --- | --- | --- |
| Thompson 1987 | Unknown | 2 |
| Polo 1993 | Unknown | 8 |
| Nielsen 1998 | SPG4 | 16 |
| Cruz 1999 | Unknown | 2 |
| Bonsch 2003 | SPG4 | 10 |
| Sartucci 2007 | SPG4 | 12 |
| Liu 2009 | SPG31 | 2 |
| Giananneschi 2014 | SPG4 | 12 |
| Geevasinga 2015 | SPG4 | 13 |
| TOTAL |  | 77 |

Table 4.2 Studies with normal upper limb CMCT in all patients studied. SPG4 (HSP-*SPAST*), SPG31 (HSP-*REEP1*).

## Studies with abnormal LL CMCT results

| **Study** | **Genotype** | **Results – prolonged CMCT** | **Results – Absent MEP** | **Results – Total abnormal** |
| --- | --- | --- | --- | --- |
| Thompson 1987 | Unknown | 2/2 (100%) |  | 2/2 (100%) |
| Pelosi 1991 | Unknown | 4/10 | 6/10 | 10/10 (100%) |
| Schady 1991 | Unknown | 12/25 | 9/25 | 21/25 (84%) |
| Polo 1993 | Unknown | 5/8 (63%) |  | 5/8 (63%) |
| Bonsch 2003 | SPG4 | 5/10 (50%) |  | 5/10 (50%) |
| Nardone 2003 | SPG4, SPG7 | 3/7 (43%) |  | 3/7 (43%) |
| Schulte 2003 | Mixed | 9/18 | 1/18 | 10/18 (56%)  SPG4 12.5%  Non-SPG4 90% |
| Klebe 2004 | SPG4 | 12/22 (55%) |  | 12/22 (55%) |
| Orlacchio 2005 | SPG4 | 11/11 (100%) |  | 11/11 (100%) |
| Sartucci 2007 | SPG4 | 11/12 | 1/12 | 12/12 (100%) |
| Orlacchio 2008 | SPG38 | 19/19 (100%) |  | 19/19 (100%) |
| Serranova 2008 | Unknown | 9/11 | 2/11 | 11/11 (100%) |
| Liu 2008 | SPG 6 | 2/6 | 4/6 | 6/6 (100%) |
| Liu 2009 | SPG 31 | 2/2 (100%) |  | 2/2 (100%) |
| Battini 2011 | SPG4, SPG31 | 14/14 (100%) |  | 14/14 (100%) |
| Manganelli 2011 | SPG 5 | 1/3 | 2/3 | 3/3 patients studied (100%) |
| Fisher 2013 | SPG 31 | 2/2 (100%) |  | 2/2 (100%) |
| Karle 2013 | Mixed | 48/128 (37%) | 46/128 | 94/128 (73%) |
| Oguz 2013 | SPG11 | 3/3 (100%) |  | 3/3 (100%) |
| Rinaldi 2015 | SPG10 | 1/1 (100%) |  | 1/1 (100%) |
| Denton 2016 | Unknown | 14/14 (100%) |  | 14/14 (100%) |
| Martinuzzi 2016 | Mixed | NS | NS | 48/49 (98%) |
| TOTAL |  |  |  | 308/377 (82%) |

Table 4.3 Studies with abnormal lower limb central motor conduction time results. SPG4 (HSP-*SPAST*), SPG31 (HSP-*REEP1*), SPG6 (HSP-*NIPA1*), SPG5 (HSP-*CYP7B1*), SPG7 (HSP-*paraplegin*), SPG11(HSP-*spatacsin*), SPG38 (HSP-*SPG38*), SPG10 (HSP-*KIF5A*).

## Studies that did not distinguish between UL and LL CMCT

| **Study** | **Genotype** | **Results** |
| --- | --- | --- |
| Sue 1997 | Unknown (5 patients) | 4/5 had B/L increased CMCT, 1/5 had absent MEP |
| Di Lazarro 1999 | Unknown (31 patients) | CMCT had 0.8 sensitivity, 0.16 false negativity compared to clinical exam (1.0 sensitivity) |
| Schule 2009 | SPG5(3 patients) | Increased CMCT, reduced amplitude for all 3 patients |
| Roos 2014 | SPG5A (3 patients) | Prolonged CCT for all 3 |

Table 4.4 Studies that did not distinguish between UL and LL CMCT. SPG5A/5 (HSP-*CYP7B1*).
